# Essential thrombocythemia

**DOI:** 10.1186/1750-1172-2-3

**Published:** 2007-01-08

**Authors:** Jean B Brière

**Affiliations:** 1 Service d'hématologie clinique, Hôpital Beaujon, Clichy, France

## Abstract

Essential thrombocythemia (ET) is an acquired myeloproliferative disorder (MPD) characterized by a sustained elevation of platelet number with a tendency for thrombosis and hemorrhage. The prevalence in the general population is approximately 30/100,000. The median age at diagnosis is 65 to 70 years, but the disease may occur at any age. The female to male ratio is about 2:1. The clinical picture is dominated by a predisposition to vascular occlusive events (involving the cerebrovascular, coronary and peripheral circulation) and hemorrhages. Some patients with ET are asymptomatic, others may experience vasomotor (headaches, visual disturbances, lightheadedness, atypical chest pain, distal paresthesias, erythromelalgia), thrombotic, or hemorrhagic disturbances. Arterial and venous thromboses, as well as platelet-mediated transient occlusions of the microcirculation and bleeding, represent the main risks for ET patients. Thromboses of large arteries represent a major cause of mortality associated with ET or can induce severe neurological, cardiac or peripheral artery manifestations. Acute leukemia or myelodysplasia represent only rare and frequently later-onset events. The molecular pathogenesis of ET, which leads to the overproduction of mature blood cells, is similar to that found in other clonal MPDs such as chronic myeloid leukemia, polycythemia vera and myelofibrosis with myeloid metaplasia of the spleen. Polycythemia vera, myelofibrosis with myeloid metaplasia of the spleen and ET are generally associated under the common denomination of Philadelphia (Ph)-negative MPDs. Despite the recent identification of the *JAK2 *V617F mutation in a subset of patients with Ph-negative MPDs, the detailed pathogenetic mechanism is still a matter of discussion. Therapeutic interventions in ET are limited to decisions concerning the introduction of anti-aggregation therapy and/or starting platelet cytoreduction. The therapeutic value of hydroxycarbamide and aspirin in high risk patients has been supported by controlled studies. Avoiding thromboreduction or opting for anagrelide to postpone the long-term side effects of hydrocarbamide in young or low risk patients represent alternative options. Life expectancy is almost normal and similar to that of a healthy population matched by age and sex.

## Disease name and synonyms

Essential thrombocythemia (ET)

Primary thrombocythemia (PT)

Hemorrhagic thrombocythemia

## Definition

Essential thrombocythemia (ET) is an acquired Myeloproliferative disorder (MPD) characterized by a sustained elevation of the platelet number with a tendency to thrombosis and hemorrhage. Elevated platelet count is related to an expansion of megakaryocytic lineage and the disorder is usually considered to be a clonal disease arising in a multipotent stem cell.

## Etiology

ET shares a molecular pathogenesis leading to overproduction of mature blood cells with other clonal MPDs such as Chronic myeloid leukemia (CML), Polycythemia vera (PV), Myelofibrosis with myeloid metaplasia of the spleen (IMF). CML is now easily recognized by the presence of the Philadelphia (Ph)-positive chromosomal abnormality and/or the evidence of a specific molecular marker, the disrupted protein kinase BCR/ABL. However, despite the recent description of the *JAK2 *V617F mutation in a subset of patients with PV, ET and IMF, the intimate mechanism underlying molecular pathogenesis of these myeloproliferative disorders is still a matter of discussion. They are therefore generally associated under the common denomination of Ph-negative MPDs. The presence of the mutation confers a proliferative and survival advantage by rendering the cells more sensitive to incoming stimulatory signals, causing clonal expansion of hematopoietic progenitors in myeloproliferative disorders [1].

## Epidemiology

The reported annual incidence rates for ET range from 0.59 to 2.53/100,000 (<9/100,000) inhabitants. A population based survey in the city of Göteborg, Sweden, adjusted to standard population reported an incidence rate of 1.55/100,000, just under the incidence rate evaluated in the same conditions for PV (1.97/100,000). The prevalence is around 30/100,000 inhabitants [2]. The diagnosis of ET is more frequently established today than in the past [2,3], the most likely explanation being a wider use of automatic count in routine examination leading to the diagnosis of more non symptomatic ET patients. The median age at diagnosis is 65 to 70 years but the range in age of onset is characteristically wide. ET is often diagnosed during the third or fourth decade of life. Since the diagnosis of ET is frequently recognized early in life and the incidence of the disease is around two times higher in females compared to males, the occurrence of ET associated pregnancies is common [4-6].

## Clinical description

The clinical presentation of ET is dominated by a predisposition to vascular occlusive events and hemorrhages.

### Vascular occlusive events

Vascular occlusive events include major thrombotic events involving the cerebrovascular, coronary and peripheral arterial circulation. Thromboses of large arteries represent a major cause of mortality associated with the disease or can induce severe neurological, cardiac or peripheral arteries disabilities.

Deep vein thrombosis also represents a potentially serious and eventually life-threatening event due to the risk of pulmonary embolism or related to the region involved as it is the case in hepatic (Budd Chiari syndrome) or portal thrombosis [7].

Vascular occlusive events can also occur in the micro-vessels where they cause a wide range of clinical symptoms, secondary to a transitory suspension of the circulation. They are caused by platelet-mediated transient occlusive thrombosis in the end-arterial circulation [8]. Aspirin-sensitive erythromelalgia, one of the most characteristic microvascular disturbances in ET, is described as burning painful and ulcerative toes. It is often accompanied by a warm, red or violet colored congested limb extremity. The ischemic attacks of digital arteries may subsequently progress towards small zones of limited necrosis or even peripheral gangrene with palpable arterial pulsations.

Headaches are the most common neurological manifestations. Their pathophysiology remains uncertain. Some of them resemble migraines. Sometimes, neurological symptoms in ET show a striking similarity with migraine aura or accompaniments. In contrast, transient typical or atypical ischemic attacks, convulsions and sudden transitory absences seem to result from an ischemic mechanism. Visual dysfunction manifests as attacks of diplopia and sudden reversible attacks of blurred vision. All these symptoms share a specific reversibility or at least sensitivity to anti-aggregating agents and occur, at least in younger ET patients, in the absence of conspicuous atheromatous lesions in the arterial system.

### Hemorrhagic manifestations

Bleeding in ET is often limited to recurrent skin manifestations: bruising, subcutaneous hematomas, ecchymoses, and epistaxis or gum bleeding. Petechiae are never seen. A history of gastrointestinal blood loss (melena and/or hematemesis) or biological evidence in favor of chronic occult blood loss may be evidenced at diagnosis. Secondary bleeding, eventually life-threatening can also occur after trauma or surgery. Hemorrhagic complications are rarely observed during the course of the disease when appropriate preventive measures are taken. Bleeding symptoms are primarily observed in patients with the highest platelet counts [9]. The bleeding diathesis is not due to impaired platelet function but rather to an acquired Von Willebrand's disease caused by proteolytic reduction of Von Willebrand Factor (VWF) multimers. There is an inverse relationship between VWF levels and platelet counts. The VWF large multimer deficiency appears at platelet counts of 1000 to 1500 × 10^9^/L and increases thereafter. Aspirin may unmask a latent bleeding diathesis and may result in severe hemorrhagic complications. It is therefore contraindicated in patients with bleeding history and a very high platelet count (in excess of 1500 × 10^9^/L leading to the acquisition of Von Willebrand's deficiency). If indicated, aspirin should be used in the widely accepted low dose regimen (100 mg daily).

### Asymptomatic presentation

The frequency of thrombohemorrhagic complications at presentation of ET varies widely in the different retrospective studies. In a group of 809 ET patients diagnosed according to the Polycythemia Vera Study Group (PVSG) criteria from 11 retrospective clinical studies [10], the incidence of thromboembolic events without bleeding was 42%, bleeding symptoms without thrombosis occurred in 1.4%, and both bleeding and thrombosis in 15% of the patients. The arterial thrombotic manifestations were described as microcirculatory disturbances in 41%. However, the most important message of this retrospective compilation was that 36% of ET patients were free of symptoms at diagnosis [11]. It is also important to note that many of them remained free of complications during the evolution of ET.

### Maternal and fetal risk in pregnancy with ET

Maternal risk is limited. The increased risk of thrombosis (present in healthy pregnant women as well) may be worsened by ET. Hemorrhagic risk is low, except in patients with acquired Von Willebrand's disease [4]. The fetus seems to be at greater risks. There is an overall increased incidence of first trimester miscarriage in one out of three pregnancies. Late pregnancy loss is far more frequently observed in ET than in normal population (5–9.6% *vs*. 0.5%). An increased incidence of intrauterine growth retardation (4–5.1%), preterm delivery (5.6–8%) and placental abruption (2.8%) was reported. Placental micro-infarctions due to increased platelet number and to platelet activation seem to be the underlying pathological basis of adverse events for the fetus. Overall live birth rate may be as low as 50 to 57% [5]. A spontaneous decrease in the number of platelets is frequently observed, beginning after the first trimester of pregnancy.

## Diagnostic criteria

Due to the lack of a specific molecular marker, the diagnosis of ET can only be settled after a step-by-step elimination of the other clinical situations associated with a protracted elevation of the platelet number. The criteria of this stepwise elimination were initially proposed by the PVSG [12]. More recently, the World Health Organisation (WHO) has proposed an improved new set of criteria where specific abnormalities of megacaryocytopoiesis associated with other bone marrow biopsy findings were proposed as a common positive marker of Ph-negative MPDs [13]. The *JAK2 *V617F mutation clearly represents a new molecular marker for the Ph-negative patients. Initially described in PV patients, where it has been observed in 65% to 97% of the cases [1,14-17], this mutation has also been detected in subsets of each of the other Ph-negative MPDs: in 23 to 57% of ET and 43 to 67% of IMF patients, as well as in some Ph-negative CML and MDS [18,19]. The presence or absence of the V617F mutation does not strictly correlate with any phenotype of Ph-negative MPD recognized according to either the PVSG criteria or the WHO classification. As the absence of the mutation has been repeatedly confirmed in more than 50% of ET patients, the diagnosis of ET presently remains a mixture of: 1) positive non specific arguments in favor of a Ph-negative MPD, including the *JAK2 *mutation and the bone marrow (BM) biopsy findings and 2) elimination of PV and IMF according to their currently used and phenotypically based definitions [12,13,20].

### Arguments supporting the diagnosis of Ph-negative MPD

• Standardized histological BM features have been introduced in the WHO diagnostic criteria for Ph-negative MPDs. The diagnostic target of histopathology in patients presenting an elevated platelet count according to this classification may be twofold:

1) **To confirm the presence of a Ph-negative MPD and exclude long lasting reactive thrombocytoses (Rth)**. This step can be achieved through a systematic analysis of: a) megacaryocytopoiesis, focusing especially on the proportion of giant *vs*. small forms, nuclear lobulation, maturation defects, cluster formation by megacaryocytes; b) BM cellularity; c) degree of expansion and of left shifting of granulocyte and erythrocyte lineages; d) densification of the reticulin network and presence of collagen fibrosis in the BM stroma [7,21,22].

2) **To offer a specific morphologic description of each of the Ph-negative MPDs**. For instance in ET, megakaryocytopoiesis is characterized by the presence of large to giant cells with a predominance of hyperlobulated staghorn nuclei loosely clustered throughout the BM. Cellularity in ET is not significantly increased compared with age-matched normal samples, and neutrophil granulopoiesis and erythropoiesis display no significant change in the distribution and proliferation of both cell lineages. In true ET there is no collagen fibrosis and no increase in reticulin fibers of the myeloid stroma [22].

The relevant parameters in BM introduced by the WHO classification to eliminate not only classical forms of PV or IMF but mainly prefibrotic early stages of IMF or/and early or latent PV will be described conjointly with the differential diagnosis of the other Ph-negative MPDs.

• The unique, acquired, clonal, somatic mutation of *JAK2 *occurring at the level of hematopoietic stem cell in a large proportion of PV patients and in a lower subset of IMF and ET patients has provided a completely new approach to demonstrate a clonal origin of the cell proliferation, in patients with a protracted platelet number. This new marker unequivocally represents a major improvement upon the previous cytogenetic studies, which were able to demonstrate clonal cytogenetic abnormalities in BM cells in less than 5% of ET patients. Interestingly, results of the direct assessment of a clonal expansion in peripheral blood cell of female ET patients with heterozygoty for X-linked genes do not strictly match the presence of the mutation. The unexpected presence of the mutation in female ET patients with non clonal hematopoiesis, according to X-linked gene studies, may only reflect a lower sensitivity of methods based on quantification of X-linked gene alleles compared to the polymerase chain reaction (PCR) detection of the *JAK2 *mutation. Absence of the V617F mutation in ET with unquestionable clonal hematopoiesis may however suggest, on the contrary, that other mutation(s) might still explain myeloid expansion in these patients [23-28].

• Prior to the description of the *JAK2 *V617F mutation, a set of positive markers including 1) "spontaneous" growth of erythrocyte precursors (EEC) in absence of added erythropoietin (EPO) [29]; 2) endogenous megakaryocytic colony formation [30]; 3) decreased expression of c-MPL at platelet or megakaryocyte cell surface; 4) polycythemia rubra vera protein 1 (PRV-1) and 5) nuclear factor erythroid 2 (NF-E2) mRNA over expression in polymorphonuclear cells were initially described in PV patients. However, like the *JAK2 *mutation, these markers were also observed in subsets of other Ph-negative MPDs, including ET. It was therefore suggested that these biological markers may be used as criteria in favor of a clonal origin for the increased platelet number observed in a given patient [31-35]. There is no longer an indication for these investigations in patients positive for the *JAK2 *V617F mutation, since it has been demonstrated that this mutation induces activation of the JAK2 kinase directly involved in the intracellular signaling, following the exposure to EPO or thrombopoietin (TPO), in the elevated expression of mRNA for PRV-1 or NF-E2 in granulocytes, or in processes involving MPL maturation and trafficking. However, since the mutation was still not found in some patients who were shown to form EEC [36] and over express PRV-1 [37], testing erythrocyte or megakaryocyte colony formation may represent a non specific alternative to BM biopsy to bring arguments in favor of an MPD in ET and PV patients without *JAK2 *mutation.

• The presence of decreased circulating EPO levels that is common to PV patients and to a subset of ET patients [38] may also be used as an argument in favor of a MPD-related origin of the elevated platelet number. However, since a strong correlation between low EPO levels and the diagnosis of PV has been demonstrated, there is a controversy over using this criterion either as a biological marker common to several kinds of MPD or as a strong indicator of PV phenotype [39,40].

## Diagnostic methods and differential diagnosis

### Platelet number

**Figure 1:**
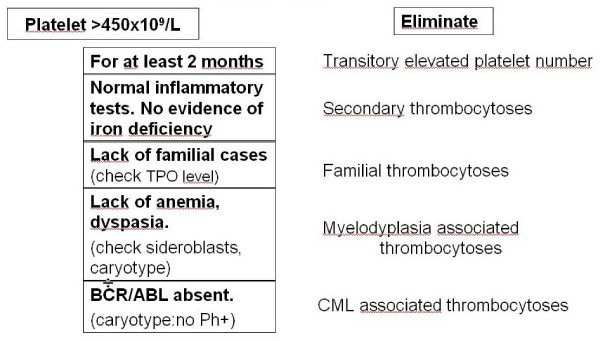
Diagnostic algorithm of thrombocytoses associated with Philadelphia (Ph) negative MPDs (Myeloproliferative disorders).

The platelet number required for considering the presence of ET has decreased since the initial definition of the disease, where it was initially fixed to over 1000 × 10^9 ^platelets/L (Figure 1). In the last version of the PVSG criteria, it has subsequently been lowered to over 600 × 10^9 ^platelets/L observed on two occasions, separated by at least one month (to eliminate a cause of transitory elevation of the platelet number). The availability of positive arguments in favor of a Ph-negative MPD-like bone marrow biopsy findings and, moreover, evidence of the V617F mutation makes it now possible to advocate this diagnosis with a platelet number just over 450 × 10^9^/L, whenever a suggestive clinical context including erythromelalgia or occurrence of arterial or venous thrombosis [7,41] is associated.

### Elimination of the causes of secondary thrombocytoses

The main causes of secondary thrombocytoses including: iron deficiency, malignancy, chronic inflammatory disease, or histories of splenectomy or of protracted marrow regeneration have to be excluded.

**Elimination of iron deficiency **includes a normal or increased serum ferritin level in the absence of inflammatory indices (erythrocyte sedimentation rate, fibrinogen, C-reactive protein (CRP)) and normal red cell mean corpuscular volume. This step remains an important one in the diagnosis of ET, both to exclude thrombocytoses related to body iron stock depletion and to recognize PV masked by iron deficiency. The original requirement for stainable marrow iron is for this reason still proposed in one of the most recent set of criteria [36]. If these measurements suggest iron deficiency, a closely monitored trial of iron therapy may be necessary to eliminate progression of hematocrit toward the polycythemic range. Again, the availability of positive arguments in favor of a Ph-negative MPD such as BM biopsy findings and, moreover, evidence of the V617F mutation, represent definitive arguments against secondary thrombocytoses (RhT), although these investigations might be required only during later steps of investigation of patients with protracted thrombocytoses.

#### Elimination of familial hereditary thrombocytoses

*TPO *gene mutations, causing elevated TPO levels and therefore leading to familial thrombocytosis, have been described [42]. The context of several cases of thrombocytoses observed in the same offspring or increased TPO levels in sporadic ET may prompt a check for a gain of function mutation of the *TPO *gene. This eventuality is different from situations where several cases of true MPDs are aggregated in the same family [43] suggesting the presence of a predisposition factor to PV, ET, IMF and CML. In these cases, additional events such as chromosome translocation (9;22) (q34;q11) in CML or several somatic mutations arising in hematopoietic stem cell (including the V617F mutation) may usually be responsible for the disease onset or for the specific characteristics of proliferation leading to the phenotypic classification of the final MPD.

#### Elimination of primary thrombocytoses associated with chronic myelodysplastic syndromes (MDS)

This step was proposed [12,13] to exclude patients whose hemograms, myelograms or cytogenetic abnormalities were suggestive of myelodysplasia. The presence of the *JAK2 *mutation in some patients with MDS and sideroblastic anemia [18,19] may justify the individualization in the most recent classification of myeloid neoplasia of a distinct myeloproliferative-myelodysplastic overlap category [44]. However, the distinction of these borderline situations from a "true ET" might still be maintained, at least in studies in which long term evolution or evaluation of treatment are concerned.

#### Elimination of primary thrombocytoses associated with the full-blown picture or with more subtle presentations of a chronic myeloproliferative syndrome different from ET

1) The elimination of the more or less pure thrombocythemic forms of CML [45] that previously led to the concept of Ph-negative MPD, is now easily achieved and based mainly on the determination of the presence of BCR/ABL transcripts in peripheral blood, rather than on the research of chromosome Philadelphia in BM cells.

### Distinction between ET and PV patient

**Figure 2:**
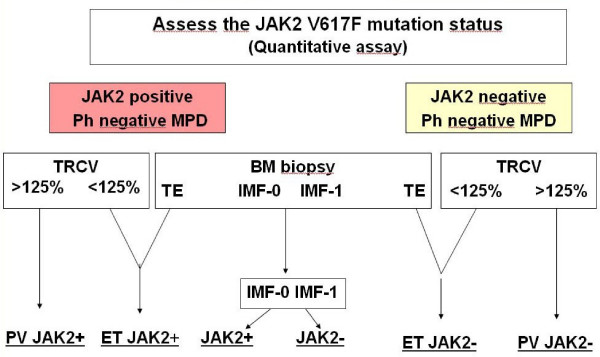
Diagnostic algorithm of Essential thrombocythemia (ET).

2) The identification of features specific to ET among patients with a primary Ph-negative MPD and an increased platelet number is more controversial. Determination of the total red cell volume (TRCV) in all patients with thrombocytoses and hemoglobin (Hb) or hematocrit (Ht) values equal or superior to normal value using a radioisotopic method [46], after exclusion or treatment of iron deficiency was recommended in the PVSG and WHO criteria for ET. Patients with TRCV>125% of normal value definitively represent PV patients with an increased platelet count (Figure 2). However, there is an unequal availability of the opportunity of TRCV measurement according to the geographical repartition of centers involved in MPD evaluations. The fact that this isotopic measurement is costly, time consuming and non specific (since, alone, it does not differentiate an increased red cell volume in congenital polycythemia (CP), secondary erythrocytosis (SE) or PV) has prompted several alternative strategies for differentiation between PV and ET. None of them can presently be regarded as universally accepted.

• Spontaneous EEC and a low serum EPO level are criteria of high specificity but low exhaustively for the diagnosis of PV. EEC assays are almost always negative in RhT and positive EEC results have been extensively reported in ET patients [40]. The presence of EEC has even been recommended as a minor criterion for the diagnosis of ET. Fifty percent of patients with ET diagnosed according to the PVSG criteria have EPO-independent EEC and a variable proportion of them have low serum EPO levels reflecting a biologically distinct subgroup of ET at risk of progression to PV [35,39].

• Standardized histological BM features (introduced in the WHO diagnostic criteria for Ph-negative MPDs) have been proposed to distinguish initial stages of PV without an obvious increase in Ht level but with an elevated platelet count, mimicking ET on the basis of an increased BM cellularity, an increased and left shifting pattern of both erythropoiesis and granulopoiesis. Megakaryocytes vary in size, generating a pleiomorphic aspect without maturation defects but with a more pronounced tendency toward clustering than in "true" ET.

• Evidence of the *JAK2 *V617F mutation (found in 90% of PV patients) has been proposed as a prerequisite criterion for the diagnosis of PV [36] but it may be of no help for differentiation of 50% of ET patients positive for *JAK2 *mutation. However, it is worth noting that differences in the phenotype presentation and clinical events between the *JAK2 *positive and *JAK2 *negative ET patients have been demonstrated [47], even if these differences are mainly statistically based and remain small. Furthermore, the presence of EEC and low serum EPO level are still observed in patients that do not carry *JAK2 *mutation [1,8]. Therefore, disease classification as well as patient management according to the *JAK2 *status seems to be premature.

### Distinction between ET and IMF patients

Patients with a full-blown picture of IMF including large spleen, tear drop shaped red cells, leukoerythroblastic blood picture and collagen fibrosis on BM biopsy are easily eliminated.

• The recognition of patients presenting an increased platelet number that may correspond to a prefibrotic phase of a Ph-negative MPD was one of the first evidences demonstrating the heterogeneity of ET defined according to the PVSG criteria. The identification of these patients is based on BM morphology according to the WHO criteria [20,48] and is associated with a significant loss of life expectancy when their evolution was compared to patients with BM feature specific of ET. Discrimination of prefibrotic or early fibrotic stages of IMF, or IMF-0 and IMF-1 respectively (as it has been proposed to name them) is based on an age-matched increase in marrow cellularity, clustering of megakaryocytes exhibiting dense hyperchromatic cloud-like nuclei and deviation of nuclear cytoplasmic maturation. This stage of prefibrotic Ph-negative MPD is characterized by a pronounced proliferation and left-shifting of the neutrophil granulopoiesis but is devoid of collagen fibrosis and even of an increase in reticulin fibers (at least in IMF-0).

• The *JAK2 *V617F mutation found in 43 to 67% of classical IMF patients is more frequently homozygous (6 to 18%) compared to ET (3 to 4%). Homozygoty for *JAK2 *mutation associated with a loss of heterozygoty on chromosome 9p is even more frequent in PV (24 to 27%) and, moreover, almost always present when PV has progressed to myelofibrosis [1,49]. In prefibrotic or early fibrotic patients (IMF-0 or IMF-1), no difference in the frequency of *JAK2 *mutation has presently been observed [47]. No data on the predictive value of the sequential evaluation of circulating CD34 cells are presently available for detection of this subset of patients whose recognition remains exclusively based on BM biopsy examination.

## Management including treatment

### Risk categories of patients with ET

Most of the published treatment algorithms [36,50-55] rely on the concept of a risk stratified management. The majority of them distinguished three risk categories based mainly on the estimation of the thrombohemorrhagic risk. This limited ambition is due to the fact that at present there is no drug known to cure the underlying disease or to prevent the risk of clonal evolution. The current rationale for using drugs is therefore either to prevent, or more rarely, to treat a thrombohemorrhagic event.

#### High risk patients

The criteria recognized as major risk of thrombosis and embolism by almost all investigators [50,51,53,55] are:

• Age: the risk increases significantly in patients older than 60 years.

• Previous thrombotic event.

When the stratification also includes an evaluation of the risk of major hemorrhage [50,51,55], the most frequently considered factors are:

• Platelet count >1500 × 10^9^/L.

• History of major bleeding or, as learned from The European Collaboration on Low-dose aspirin in Polycythemia Vera (ECLAP) experience, a history of minor bleeding with a platelet count >1000 × 10^9^/L, especially in patients with a long disease duration >15 years) [56].

#### Low risk patients

The most restrictive definition of patients at low risk includes:

• Patients aged less than 40 years.

• Patients presenting with no high risk feature.

• Patient without cardiovascular risk factor and thrombophilia of clinically significant familial expression.

#### Intermediate risk patient

Intermediate risk patients represent a category without a clear consensus on its definition. The risk of thrombosis is often regarded as intermediate according to:

• Age (patients aged between 40 and 60 years).

• Well established risk factors of cardiovascular disease (hypertension, diabetes, smoking and hypercholesterolemia). In the Medical Research Council (MRC) PT1 study the presence of these factors was even sufficient to include patients in the high risk group.

• An intermediate risk related to a platelet count between 1000 and 1500 × 10^9^/L has also been proposed [50,56]. In this case, the platelet number was regarded as increasing the thrombotic risk when associated with a vascular risk factor or familial thrombophilia [50], and the hemorrhagic risk when associated to either minor bleeding or long term duration of the disease [56].

### Therapeutic intervention in ET patients

To reduce the vascular risk in ET patients (except for the management of reversible cardiovascular risk factors) the therapeutic intervention is limited to the decision of decreasing the platelet count or altering the platelet function by aspirin or an equivalent therapy.

### Therapeutic options

As a first step in their management, patients should have an assessment of their risk factors for vascular disease and, if necessary, modulation of hypertension, hyperlipemia, smoking habits and overweight.

#### A. Anti-platelet treatment

##### Aspirin (and alternative anti-platelet agents)

Aspirin (Asp) is the standard therapy for ischemic manifestations of the microcirculation. Reducing the number of platelets also attenuates the symptoms. Continuous Asp therapy is indicated when the occlusive manifestations of the microcirculation persist despite lowering the number of platelets.

The role of Asp in the prevention of arterial thromboses has been clearly established in the general population. The benefit of a low dose Asp in preventing the risk of thrombotic complications without increasing significantly the hemorrhagic risk in PV patients has recently been demonstrated in the extensive prospective ECLAP study [57]. A preliminary pilot study in polycythemia patients receiving either 40 mg/day of Asp or a placebo showed that this dosage fully inhibited the cyclooxygenase activity and did not cause any major hemorrhage [58]. Anti-platelet therapy has not yet prospectively been shown to reduce the incidence of thrombosis in ET, however, the combination of an anti-aggregating agent with cytoreductive therapy has been found to be safe and to reduce the incidence of thrombosis in ET patients in retrospective studies [59,60]. Low dose Asp is often prescribed as soon as an excess of platelets is discovered by the general practitioners, however, objective data in favor of the preventive role of Asp against the vascular risk in symptom-free patients not taking a platelet-lowering treatment are not presently available and the use of Asp is contraindicated in ET patients with extreme thrombocytosis in whom the presence of Acquired Von Willebrand syndrome might be screened.

***ADP receptor antagonists ***(thienopyridines such as ticlopidine and clopidrogel) have a growing role in patients with ongoing thrombotic events (suggesting resistance to Asp). At present, there are no data to recommend their use in MPD patients [61].

#### B. Platelet-lowering treatment

Hydroxyurea (HU) has emerged as the reference platelet-lowering agent in high risk ET patients. A valuable platelet lowering as well as anti thrombotic activity has been demonstrated in both of the (only) two published randomized prospective trials concerning the treatment of this category of patients [62,63]. However, the still ongoing controversy (according to whether or not HU is leukemogenic) has prompted the consideration of two main alternative drugs: anagrelide, now authorized for marketing in European countries, and interferon **α **(IFN-**α**). Two more medications, pipobroman (available only in certain European countries) and busulfan are also used as platelet-lowering drugs in ET patients, as well as platelet apheresis, which may be the preferred therapeutic option in case of emergency.

##### Hydroxyurea (Hydroxycarbamide, HU)

###### Efficacy

The efficiency of HU in controlling the platelet number in high risk ET patients has been further documented by the recently published results of the MRC PT1 trial [63]. A lack of platelet control was observed in less than 4% (15 patients) of the 404 patients treated by HU. At 3 months 90% of the patients had a platelet number <600 × 10^9^/L. The median platelet count at 6 months was <400 × 10^9^/L. Stable reduction of the median platelet number (lower or equal to 400 × 10^9^/L) was obtained with HU for the following 24 months. Importantly, the protection from thrombosis in high risk ET patients already demonstrated by Cortellazzo *et al*. with HU (plus anti-aggregating agents in almost 70% of the patients) [62] was confirmed by this study with HU associated with Asp in all the patients. After 2 years, the prevalence of thrombotic events was 4%, identical to the former study of Cortelazzo, and significantly lower than the prevalence of 24% found in the control group [62].

###### Side effects

Clinical and hematological tolerance of HU is usually good even during very long periods of time. Major short term toxic effects are dose limiting hematological impairment and fever. However, oral and leg ulcers and other skin lesions are currently observed but often only after several months or years of treatment, suggesting the role in their occurrence of the cumulative dose received.

###### Mutagenic potential and long term resistance

HU was initially introduced in the treatment of ET patients because it is supposedly non mutagenic. Its mechanism of action is the inhibition of DNA synthesis by blocking the ribonucleoside reductase activity. As leukemogenic effects of HU have not been definitively eliminated, this molecule should be used cautiously in young individuals. After a continuous use in ET patients, the non selective effect of the drug on platelet reduction may, in the long run decrease, lead to anemia and neutropenia after dosage escalation of HU. This delayed hematological toxicity (leading to a withdrawal of the drug) sometimes seems to be related to a progression of the disease.

A unified definition of the clinical resistance and intolerance to HU in ET is proposed [64] and includes:

1. A platelet count persistently high (>600 × 10^9^/L) after 3 months of treatment by HU at maximum daily possible dosage.

2. Or at any dose of HU: a) A decrease of the white blood cell (WBC) counts under 2.5 × 10^3^/mm^3 ^or of Hb level under 10 g/dl to maintain the platelet number under 400 × 10^9^/L; b) Presence of leg ulcers or other unacceptable muco-cutaneous manifestations; c) Presence of HU related fever. In these situations the risk of leukemic progression in patients requiring treatment by multiple agents should not be underestimated and has implications for the choice of a second agent [51].

The status of ET patients according to the *JAK2 *V617F mutation might affect the response to HU treatment. A recent study of 640 high risk patients (MRC PT1 trial, *JAK2 *genotype data available) has demonstrated that HU-treated patients positive for the *JAK2 *mutation had a persistently lower platelet count than the individuals negative for the *JAK2 *mutation. Furthermore, ET patients positive for the *JAK2 *mutation were more sensitive to HU than ET patients without this mutation, as demonstrated by lower doses HU required to control their platelet count and higher decrease in their Hb concentration and WBC counts compared to the pre-treatment levels. These differences in treatment sensitivity according to the mutation status of high risk ET patient was not seen in the group treated with anagrelide (see below) [47].

##### Anagrelide

Anagrelide (Ana) is an oral imidazoquinazolin compound initially developed for its potent inhibition of platelet aggregation. It causes thrombocytopenia at doses below those exhibiting an anti-aggregating effect.

###### Efficacy

In the largest study to date, the efficacy of anagrelide in controlling the platelet count was evaluated and demonstrated in 934 ET patients [65]. This was further documented in high risk ET patients by the recently published results of the MRC PT1 trial [63]. In this prospective randomized study, a lack of platelet control was observed in less than 4% (19 patients) of the 405 patients treated by anagrelide. At 3 months the 90th percentile of platelet count was only <800 × 10^9^/L. However, at 6 months the median platelet count was <400 × 10^9^/L and a stable reduction of the median platelet number (lower or equal to 400 × 10^9^/L), similar to what was obtained with HU, was maintained during the following 24 months. A mechanism involving a diminution of the size and ploidy of BM megacaryocytes without modification of their number is supposed at the origin of the platelet number reduction. This may explain why anagrelide does not usually modify the number of white cells or does not cause a significant reduction of the Hb concentration.

The comparison of HU+Asp *vs*. Ana+Asp in this study restricted to high risk patients [63] has demonstrated that anagrelide and aspirin offer a confirmed protection from thrombosis. The prevalence of thrombotic events for anagrelide-treated patients was 8% (significantly less than 24% in the control study arm, without cytoreduction of the princeps control study of Cortelazzo *et al*.) [62]. However, in this trial (despite an equivalent control of the platelet number) protection from thrombosis in patients treated by Ana+Asp was less efficient than with HU+Asp (prevalence of thrombotic events 4% at 2 years). Interestingly, protection from arterial thrombosis and especially AIT (transient ischemic attack) was better with HU+Asp; high risk ET patients treated with Ana+Asp had a lower rate of venous thromboembolism. In the same study but in an article published later [47], it was observed that the rates of arterial thrombosis in *JAK2 *V617F positive patients randomized to anagrelide were higher than in those randomized to HU, whereas equal numbers of arterial thromboses were observed in the two arms of treatment for the patients negative for *JAK2 *V617F mutation.

###### Side effects and mutagenic potential

To date, 2251 patients were evaluated for safety of anagrelide [65]. With a maximum follow-up of 7 years, leukemogenic effect has not been demonstrated. Major side effects of anagrelide are consistent with its mechanism of action as a phosphodiesterase inhibitor [51] and include headache (12.6%), tachycardia-type palpitations (15.5%), fluid retention (6%) and gastrointestinal intolerance (14%) leading to premature interruption of the treatment in 22% of the patients [63]. A small number of reversible cardiomyopathies have been described, suggesting that an evaluation of cardiac function should be performed in elderly patients with previous cardiac disorders [53].

The comparative study of HU+Asp *vs*. Ana+Asp of the MRC PT1 trial has shown that anagrelide was more poorly tolerated than HU (frequency of side effects 21.7% *vs*. 10.6%). The drop-out rate has been estimated to around 14% [66]. There was more concern in this study from the statement that major hemorrhages occurred more frequently in the Ana+Asp arm than in the HU+Asp arm, suggesting a potentiation of the anti-aggregating effects of aspirin by its association with anagrelide. The question raised by the difference in the rate of progression toward myelofibrosis observed in the anagrelide arm (16 *vs*. 5 events) needs further evaluation. A previous study comparing BM biopsy findings before and after therapy failed to demonstrate a progression in reticulin or collagen fibrosis during the treatment with anagrelide [67]. Further data may be expected from an on-going study comparing anagrelide to HU in ET patients (newly diagnosed according to the WHO criteria) in whom BM findings will be submitted to a panel revaluation (Study by AOP pharma 007).

Anagrelide has been approved by Food and Drug Administration (FDA) and by European authorities as oral treatment for ET and thrombocytoses associated with PV. Nevertheless, its usage is recommended in case of failure or intolerance to HU.

##### Interferon-α (IFN-α)

###### Efficacy

Efficiency of IFN-**α **administered by subcutaneous injections to reduce the platelet number has been documented in a large number of cohort studies [50]. Only a preliminary report of a multi-center comparative (HU *vs*. IFN) randomized trial has been published [68]. The overall response rate was 84% and a complete normalization of the platelet count was obtained in 53% of the patients after 3 months. A positive effect on clinical symptoms was associated with normalization of the platelet count; precise information about the reduction of the rate of thrombotic events in high risk patients is not presently available [69].

###### Side effects and mutagenic potential

No associated leukemogenic effect has been reported with IFN-**α**. Side effects, mainly flu-like syndrome, are common at the initiation of IFN treatment. They can subside thereafter, mainly when IFN doses are sharply lowered during the subsequent maintenance therapy. However, they lead to discontinuation of the drug in 16.5% of the patients.

Interesting data from phase II studies of pegylated (Peg) IFN-**α **are now available. The rate of subcutaneous injections, only once per week, improves the compliance to the treatment and increases the number of long lasting hematological responses [70]. A preferential targeting of the *JAK2 *V617F mutated clone by Peg-IFN-**α **has been recently suggested by a phase II study in PV [71]. In this prospective study of 27 PV patients positive for the *JAK2 *mutation, a high rate of complete hematological responses was observed together with a decreased expression of mutated *JAK2 *in peripheral blood cells in 87% of patients.

#### C. Alternative cytoreductive therapy

##### Pipobroman (PI)

Pipobroman (PI) is a piperazine derivative. PI appears to competitively inhibit pyrimidines though it shows a structural resemblance to alkylating agents.

###### Efficacy

The efficacy of PI in controlling the platelet number in patients with ET is close to that of HU, with an overall response around 95%, documented in several cohort studies [72-74]. In 118 ET patients at high risk for thrombosis treated by PI, protracted hematological remissions and good clinical and hematological tolerances have been reported after a long follow-up [74]. The cumulative risk of thrombosis at year 10 was equal to 14%. Therefore, although not demonstrated by prospective or comparative studies, PI has presumably, like HU, a role in preventing thrombotic events in high risk patients.

###### Side effects and mutagenic potential

Treatment with either HU or PI was carried out on randomized 292 patients with PV [75]. The risk of leukemia was approximately 10% after13 years of follow-up, with no significant difference between the two arms. Therefore, a long term leukemogenic risk was suspected in PI-treated PV patients, even if this treatment was given alone [75]. In ET, however, the cumulative risk of acute leukemia was 3% at 10 years and 6% at 15 years [73]. A comparison, although retrospective, between PI and HU has been recently assessed. After a median follow-up of 104 months in a cohort study of 155 ET patients, a significantly lower transformation rate was observed in PI-treated patients [76].

##### Busulfan (BU)

Busulfan (BU) is an alkylating agent formerly used in the treatment of chronic myelocytic leukemia. Despite the reputation of this drug to induce severe and protracted bone marrow aplasias in CML, BU is sometimes proposed to control the platelet number in elderly patients with ET. Rationale for this usage comes from the statement that it is the only treatment able to induce sustained hematological remissions without need of maintenance therapy.

### Treatment algorithm

#### All patients

• Check and treat all the reversible cardiovascular risk factors (hypertension, hypercholesterolemia, diabetes, obesity, smoking).

• Consensus to recommend either a systematic investigation of associated constitutional or acquired factors of thrombophilia in patients without a clinically significant familial history or to screen average ET patients for Acquired Von Willebrand syndrome is lacking.

#### Hight risk patients

• Cytoreduction is recommended in high risk patients.

In the older patient group (>60) the recommended cytoreductive treatment is HU. In case of resistance or intolerance to HU, consider only non-mutagenic alternative drugs for second line treatment.

There is still reluctance in using HU in young patients. High risk patients under 60 must be informed of the potential side effects occurring after a protracted use of the drug. Non-mutagenic alternative (anagrelide or IFN-**α **may be considered, although antithrombotic effects of these drugs are regarded presently either as inferior to HU (in term of AIT or arterial thrombosis (for anagrelide) or not yet evaluated (for IFN).

◦ The aims of cytoreduction are to bring the platelet number into the normal range in patients with high risk of thrombosis. In patients whose high risk is mainly hemorrhagic, lowering the absolute platelet number largely under 1000 × 10^9^/L may be the most important goal.

◦ Aspirin therapy is recommended in high risk patients (except when contra-indicated by platelet count >1500 × 10^9^/L, history of major bleeding or presence of three minor risk factors). The benefit of combining of anti-aggregating agent with a cytoreductive therapy (although not demonstrated in randomized studies in ET) is supported by the demonstration of antithrombotic effect of this combination in PV patients and by the presumptive thrombotic role of platelet activation.

#### Low risk patients

• Abstention of cytoreduction is recommended in low risk patients. The risk of thrombosis in asymptomatic low risk ET patients has been evaluated in several studies [77,78] and may not be high enough to warrant the use of potentially mutagenic or toxic cytoreductive therapy [36,50,53].

• Prescription of low dose aspirin is often proposed [36,53] and may rely again on the presumptive role of platelet activation in vascular risk.

#### Intermediate risk patients

• Consider cytoreduction in patients in the intermediate age group (40–60), if associated risk factors are present (uncontrolled cardiovascular risk factor, thrombophilia of significant familial expression).

• Prescription of low dose aspirin is supported in this group of patients by uncontrolled observational studies in ET patients.

### Management of ET in pregnancy

Information available in the medical literature for the management of ET in pregnancy is still limited, but a large number of pregnancies have been reported in retrospective studies [4-6]. Recommendations for management of ET in pregnancy based on current knowledge have been elaborated [5]. They include an evaluation of the maternal risk of thrombosis and hemorrhages, based on the current characteristics of the disease: previous venous or arterial thrombosis and previous hemorrhages (during previous pregnancies or not), as well as ET induced previous complications: early (first trimester) or late (second or third trimester) pregnancy loss, intrauterine death or still birth, decreased birth weight (of more than 5th centile for gestation), severe pre-eclampsia, ante- or post-partum hemorrhage. Evaluation of the trend in the platelet number in patients without current cytoreduction is mandatory. The threshold for platelet reduction may be over 1500 × 10^9^/L rather than over 1000 × 10^9^/L due to expected natural decline of platelets after the first trimester of pregnancy. A systematic biological screening for associated prothrombotic risk factors is not clearly indicated in patients without personal or familial history of thromboses. Strong coordination between the obstetrician (monitoring the pregnancy) and the hematological department (managing ET) is mandatory.

The therapeutic options include antithrombotic treatment and cytoreductive agents. Low dose aspirin is safe during pregnancy. However, demonstration of a significantly better outcome of pregnancies evolving under continuous aspirin treatment has not been confirmed by a recent report [6]. Some studies have suggested that aspirin from the onset of pregnancy plus heparin from the second trimester might improve pregnancy outcome [79].

Many pregnancies in ET have a successful outcome with minimal therapy. Cytoreductive treatment should preferably be avoided during pregnancy, especially during the first trimester. None of the agents currently used for cytoreduction in ET has a product license for use in pregnancy. As IFN-**α **does not cross the placental barrier, it is a potential therapeutic solution when platelet reduction is required for high risk ET pregnant patients. A small number of publications is available, showing, up to now, no toxicity for the fetus [5,80]. Breast feeding is however not recommended during cytoreductive treatment including IFN-**α**. Several retrospective analyses have also led authors to suggest that reducing the platelet number using IFN-**α **for patients with prior pregnancy events might improve the chance of a successful outcome in subsequent pregnancies.

When ET has been already diagnosed, some important issues concerning the risk of pregnancy should be discussed before conception. For ET female already treated with HU, a wash-out period of 3 months has been recommended. However, for pregnancies where HU had been used during a more or less limited period of time, the toxicity of the product is probably less than it might be anticipated.

Anesthetists prefer aspirin to be stopped two weeks before delivery, to prevent the risk of hematoma after epidural and spinal anesthesia. Low molecular weight heparin (LMWH) is given as an alternative to aspirin. The greatest risk for thromboembolism is *post partum *and prophylaxis, usually in the form of aspirin and LMWH, should be continued for several weeks.

## Prognosis

No substantial differences between life expectancy of 247 patients with ET, according to the PVSG criteria and that of a control population were found by Rozman *et al*. [76,81]. Nevertheless, the common feeling that ET is a rather benign MPD, affecting more the quality of life than the overall life expectancy, has been challenged by recent publications. An eventual loss of life expectancy may be demonstrated if the group of observed patients is chosen according to selective BM based criteria. Discrimination of prefibrotic (IMF-0) or early fibrotic (IMF-1) stages of IMF from true ET, according to WHO classification, demonstrated a shortening of life expectancy of 32.3% and 21.6% for the first two subgroups compared to only 8.9% for true ET patients [22]. Bazzan *et al*. compared a cohort of 187 consecutive ET patients to age-matched healthy people living in the same area during a follow-up period of 15 years [82]. This study demonstrated a significantly higher mortality rate and a lower thrombosis-free survival in patients younger than 55 years. On the contrary, in a group of 74 women with ET younger than 50 years at diagnosis, the overall survival was similar to that of an age- and sex-matched control population, with a low incidence of life-threatening thrombohemorrhagic complications or acute leukemia but an increased incidence of first trimester miscarriages [83].

Due to the heterogeneity of the disease, suggested by these prognostic discrepancies, efforts toward understanding the risk of hemostatic complications in ET patients, as well as the risk factors involved in the clonal progression toward PV, IMF, myelodysplasia and acute leukemia have been attempted for a long time.

### Risk of thrombosis

The Cortelazzo study [84] in a cohort of 100 historical patients with ET has shown that the overall risk of thrombosis was equal to 6.6 % patients/year compared to 1.2% control individuals/year. Three important risk factors were identified by this study. The risk of thrombosis increases with age (1.7% patients/year before 40 years; 6.3% patients/year between 40 and 60; 15.1% patients/year after 60 years). A previous history of vascular occlusive episode increases the incidence of thrombosis from 3.4 to 31.4% patients/year. Patient's exposure time to elevated platelet number was also, in this study, predictive for the risk of thrombosis, however no document has ever demonstrated proportionality between the thrombotic risk and the degree of elevation of the platelet number. Moreover, 10 to 20% of severe thrombotic complications occur in ET patients with a platelet number under 600 to 700 × 10^9^/L [85].

A second study by Cortelazzo *et al*. demonstrated that the reduction of the platelet count by HU randomized against abstention of cytoreductive treatment in a group of 114 ET patients with an elevated risk for thrombosis (age over 60 or previous thrombotic event) resulted in a significant prevention of thrombosis or severe ischemia (AIT) even in the background of an anti-aggregation therapy administered in nearly 70% of patients [62].

A number of other features have been claimed to correlate with thrombotic complications in ET:

• The presence of thrombophilia: an increased risk of venous thromboembolism has been associated with a carriership of factor V Leiden [86]; an increased prevalence of antiphospholipid antibodies in ET patients has been also noted [87].

• Cardiovascular risk factors such as hypertension, smoking and hypercholesterolemia have been implicated in the increased risk of thrombosis in ET patients, as shown by retrospective studies [88-90].

• Presence of the *JAK2 *V617F clonal mutation and platelet and granulocyte activation pattern are still subject to investigations.

Clonal pattern of X chromosome inactivation [25-27], reduced expression of MPL in BM megakaryocytes and over expression of PRV1 mRNA in peripheral blood granulocytes [34,35] (previously proposed as criteria in favor of the diagnosis of clonal MPDs) were all associated with an increased risk of thrombosis. However, the role of the clonal mutation of the *JAK2 *tyrosine kinase gene in the risk of thrombosis is still controversial. The presence of the mutation may increase the risk of arterial thrombosis [91], may be associated with a global increase of vascular complications [1] or may correlate, but not to a significant level, to the elevated risk of venous thrombosis at diagnosis [47]. In another study, however, the *JAK2 *mutation status had not been correlated to the occurrence of thrombotic events [92].

The role of platelet hyperactivation [93] and granulocyte activation pattern [94] in the risk of vasculo-occlusive episodes in ET patients have been previously suggested. In more recent studies, a *JAK2 *gene dosage effect on granulocyte activation pattern was demonstrated in MPDs [49] along with, in ET, the activation of monocytes and platelet being more frequently observed in patients with a history of thrombosis [95]. In this last study, the *JAK2 *V617F mutation was found associated with the increased platelet activation. Clearly more information is needed regarding gene mutations and peripheral cells activation and their incidence on vasculo-occlusive complications.

### Hemorrhagic risk

A risk of major hemorrhage of 0.33% patients/year during follow-up has been deducted from 21 previously published studies [50]. As already mentioned, severe hemorrhages were reported to be more common in patients with platelet count >1500 × 10^9^/L but the risk decreases when platelet number is reduced by treatment, allowing the use of low dose aspirin associated to cytoreduction in these patients. In the ECLAP study of PV patients, a history of previous bleeding problems was also an important predictor of major hemorrhagia. Recently, a tentative categorization of the hemorrhagic risk has been proposed [56]:

A) **High**: in patients with platelet number >1500 × 10^9^/L or history of major bleeding or presence of all three minor risk factors. Minor risk factors proposed are: 1) Disease duration over 15 years; 2) Platelet count >1000 × 10^9^/L; 3) History of minor bleeding.

B/**Intermediate**: when patients have two minor risk factors.

C/**Low**: in all the other cases.

### Risk of a clonal progression of ET

#### • progression or transformation into PV

In ET patients, an increase of the Hb and Ht levels toward values observed in patients with primary erythrocytosis, or even a progression to a full-blown picture of PV (according to the PVSG criteria) has been observed in up to 5–6.5% of large series of patients [96]. A critical revue of the criteria used at diagnosis to differentiate ET and PV may probably explain some of these apparent early progressions. The presence of EEC in BM cells, an increase level of PRV-1 mRNA in granulocytes and low circulating EPO levels in ET patients has already been proposed as an indication of a close relationship between PV and a subset of ET patients [35,38]. More recently, in a large prospective study of 806 ET patients it has been observed that progression to PV was restricted to patients with the *JAK2 *V617F mutation [47]. In this study, statistically higher Hb and circulating granulocytes levels, lower EPO values and hypercellular BM on biopsy material in *JAK2 *V617F positive-compared to *JAK2 *negative ET patients, suggested a continuum in mutated patients between ET and PV phenotypes. A statistically different rate of progression to a PV phenotype in *JAK2 *mutated ET patients was also observed in a retrospective study with a long term follow-up [92].

#### • progression into myelofibrosis

The risk of myelofibrotic transformation is shared by all Ph-negative MPDs diagnosed according to the PVSG criteria. Myelofibrosis itself is not a disease but rather a reaction pattern of the BM to cytokines released from the clonal proliferative cells in PV and IMF [97]. In ET (according to the PVSG criteria) myelofibrosis is a delayed event with a cumulative risk of occurrence estimated at 3% after 5 years, 8% after 10 years and 15% after 15 years [98]. No clear predictive factor of the risk in patients diagnosed according to the PVSG criteria has been published. According to the WHO classification based on BM biopsy findings, the risk of fibrotic progression has been estimated to 50% after a median follow-up of 38+/-30 months in IMF-0 or IMF-1 patients. In the description of IMF-0 and IMF-1, the degree of megakaryocyte dysplasia and the level of granulocyte hyperplasia are important features and may confirm their role in the risk of fibrotic progression. Fibrotic progression might rarely and even never be observed in true ET patients [22,48]. In fact, this point needs confirmation coming from long term prospective studies.

The status of the *JAK2 *mutation, however, was not correlated with the presence of fibrosis in ET patients included in the large prospective MRC PT1 study. In PV patients, although the role of *JAK2 *V617F mutation in progression toward fibrosis is currently unknown, transition from heterozygoty to homozygoty for *JAK2 *may represent an important step in the progression toward post-PV myelofibrosis [49].

#### • Progression to acute leukemia

In the published literature (concerning often small series of ET patients with different lengths of follow-up), the risk of progression to acute leukemia or myelodyplasia during follow-up ranges from 0.6 to 6.1% [99]. Progression to acute leukemia represents the most serious life-threatening complication of ET but is always a late occurring event, with a median delay of occurrence of 6.5 years [99-101]. The predicting factors of leukemic progression risk include cytogenetic abnormalities, previous progression to myelofibrosis or treatment by cytotoxic agent(s) [101].

Untreated patients can transform, but only very rarely, into acute leukemia [99]. A retrospective study of 2316 Italians suggested an incidence of approximately 1% [102]. Patients who received previous therapy with P32 or alkylating agents, or who require more than one cytotoxic agent, have an increased risk of acute myeloid leukemia (AML)/MDS [100-105]. It is not clear whether this reflects the combined effect of the drugs (including HU, which is the most frequently used drug) or the selection (through the necessity of more aggressive treatment modalities) of more evolved and, hence, more progressive forms of the disease.

However, the leukemogenic potential of HU used alone is still a matter of controversy. Prospective randomized studies in ET patients [63,104,105] did not find significant difference in the incidence of acute leukemia between untreated patients, or patients treated by HU or anagrelide alone. However, randomized studies either lack of the long term follow-up (necessary to evaluate the late toxicity of HU) or were conducted according to the PVSG criteria (ignoring the distinction between IMF-0 and IMF-1 and true ET). According to the WHO classification, IMF-0 and IMF-1 are, in fact, early IMF with a higher propensy to leukemic transformation than the true ET. In a large retrospective study of 357 ET patients treated with HU (most of them unbiopsied), progression to AML/MDS was observed in 4.5%, frequently associated with deletion of 17p [101]. In the same study (201 ET patients who had been treated only with HU) the rate of progression to AML/MDS was 3.5%. Nielsen and Hasselbalch [106] reported progression to AML/MDS in about 3.4% in HU-treated ET patients. The most relevant information of leukemogenicity of HU is expected to come from prospective randomized studies of patients classified according to the WHO guidelines.

Information about the risk of progression to leukemia and the *JAK2 *mutation status of ET patients is still limited. No correlation has been demonstrated. Moreover, the study of blast cells in two *JAK2 *mutated ET patients with acute leukemia failed to demonstrate the *JAK2 *mutation in one of them [107].

## Unresolved question

### Implication of the description of *JAK2 *V617F mutation on the management of ET

Data on the *JAK2 *mutation status of ET patients cannot, at present, be used for prognosis assessment. Correlations between the *JAK2 *status and the vascular risk and eventuality of clonal progression remain to be established. For the same reasons, a therapeutic approach based on the *JAK2 *positive or negative status of an individual patient has presently no clinical relevance. However, future prospective studies regarding the treatment of ET must include a quantitative approach of *JAK2 *V617F expression as well as a sophisticated interpretation of the bone marrow findings.

### Relevance of therapeutic strategies aiming at reduction or eradication of the clonal disorder

Allogeneic bone marrow transplantation is exclusively discussed for those patients who have transformed into acute leukemia or myelodyplasia or have progressed into high risk myelofibrosis. Even in this context, transplantation should be regarded as experimental therapy [108]. At variance with the model proposed in CML, where a control of the BCR/ABL positive clone is now the goal of treatment, data concerning the incidence of a similar therapeutic approach in ET are lacking. The expected availability of new tyrosine kinase inhibitors may offer opportunities to test this strategy.

## Abbreviations

AIT = Transient ischemic attack

Ana = Anagrelide

AML = Acute myeloid leukemia

Asp = Aspirin

BM = Bone marrow

BU = Busulfan

CML = Chronic myeloid leukemia

CP = Congenital polycythemia

CRP = C-Reactive protein

ECLAP = European Collaboration on Low-dose Aspirin in Polycythemia Vera

EEC = Endogenous Erythrocyte Colony formation by erythrocytes progenitors

EPO = Erythropoietin

ET = Essential thrombocythemia

Hb = Hemoglobin

Ht = Hematocrit

HU = Hydroxyurea

INF = Interferon-**α**

IMF = Myelofibrosis with myeloid metaplasia of the spleen

LMWH = Low molecular weight heparin

MDS = Myelodysplastic syndrome

MPD = Myeloproliferative disorder

MPL = Thrombopoietin receptor

MRC PT1 = Medical Research Council PT1 study

NF-E2 (Nuclear factor erythroid 2)

PCR = Polymerase chain reaction

PI = Pipobroman

PRV-1 = Polycythemia rubra vera protein 1

PT = Primary thrombocythemia

PV = Polycythemia vera

PVSG = Polycythemia Vera Study Group

RhT = Secondary thrombocytoses

Rth = Reactive thrombocytoses

SE = Secondary erythrocytosis

TPO = Thrombopoietin

TRCV = Total red cell volume

VWF = Von Willebrand factor

WBC = White blood bell

WHO = World Health Organization
